# Asymptomatic and sub-microscopic malaria infection in Kayah State, eastern Myanmar

**DOI:** 10.1186/s12936-017-1789-9

**Published:** 2017-04-04

**Authors:** Myo Thiha Zaw, Myo Thant, Tin Maung Hlaing, Naing Zin Aung, Min Thu, Kanit Phumchuea, Kanokwan Phusri, Teerawat Saeseu, Ritthideach Yorsaeng, Wang Nguitragool, Ingrid Felger, Jaranit Kaewkungwal, Liwang Cui, Jetsumon Sattabongkot

**Affiliations:** 1 Defence Services Medical Research Centre (DSMRC), Nay Pyi Taw, Myanmar; 2 Loikaw Military Hospital, Loikaw, Kayah Myanmar; 3 Mahidol Vivax Research Unit (MVRU), Faculty of Tropical Medicine, Mahidol University, Bangkok, Thailand; 4 Department of Molecular Tropical Medicine and Genetics, Faculty of Tropical Medicine, Mahidol University, Bangkok, Thailand; 5 Department of Medical Parasitology and Infection Biology, Swiss Tropical & Public Health Institute, Basel, Switzerland; 6 Department of Tropical Hygiene, Faculty of Tropical Medicine, Mahidol University, Bangkok, Thailand; 7 Department of Entomology, The Pennsylvania State University, 501 ASI Building, University Park, PA 16801 USA

**Keywords:** Asymptomatic, Sub-microscopic, Malaria, Myanmar

## Abstract

**Background:**

Myanmar has the heaviest burden of malaria in the Greater Mekong Sub-region. Asymptomatic *Plasmodium* spp. infections are common in this region and may represent an important reservoir of transmission that must be targeted for malaria elimination.

**Methods:**

A mass blood survey was conducted among 485 individuals from six villages in Kayah State, an area of endemic but low transmission malaria in eastern Myanmar. Malaria infection was screened by rapid diagnostic test (RDT), light microscopy and real-time polymerase chain reaction (PCR), and its association with demographic factors was explored.

**Results:**

The prevalence of asymptomatic *Plasmodium* spp. infection was 2.3% (11/485) by real-time PCR. *Plasmodium vivax* accounted for 72.7% (8/11) and *Plasmodium falciparum* for 27.3% (3/11) of infections. Men were at greater risk of infection by *Plasmodium* spp. than women. Individuals who worked as farmers or wood and bamboo cutters had an increased risk of infection.

**Conclusion:**

A combination of RDT, light microscopy and PCR diagnostics were used to identify asymptomatic malaria infection, providing additional information on asymptomatic cases in addition to the routine statistics on symptomatic cases, so as to determine the true burden of disease in the area. Such information and risk factors can improve malaria risk stratification and guide decision-makers towards better design and delivery of targeted interventions in small villages, representative of Kayah State.

## Background

Myanmar has the heaviest burden of malaria among the Greater Mekong Sub-region (GMS) countries. Although the long-term trend showed decreasing malaria morbidity and mortality in Myanmar from 2001 to 2013 [1], there were still 152,195 confirmed malaria cases in 2013, resulting in 92 deaths [2, 3]. In addition, Myanmar is the major source of malaria introduction to its neighboring countries, which impedes progress toward the goal of regional malaria elimination by 2030. Moreover, with the rise of artemisinin resistance in *Plasmodium falciparum*, continued political, financial and scientific commitment is required if the ambitious goal of malaria elimination is to be realized [4].

Asymptomatic malaria infections are usually undetectable and rarely treated. As a major source of gametocytes for local mosquito vectors, such infections contribute to the persistence of malaria transmission [5–10]. Significant proportions of asymptomatic infection have been reported in malaria-endemic countries in the GMS [11–17]. This means that eliminating this reservoir of *Plasmodium* parasites in asymptomatic carriers using strategies such as mass drug administration may play a critical role in the elimination of malaria [18].

Despite recent improvement of malaria surveillance in Myanmar, the current data on malaria only represent symptomatic cases, which may be the ‘tip of the iceberg’ of the entire infected population. In addition, because symptomatic cases have mostly been detected by microscopy and rapid diagnostic test (RDT), which have limited detection sensitivity, patients with low-density parasitaemia have likely been missed by these surveys. This study aimed to determine the malaria situation in an eastern state of Myanmar, where malaria has been highly endemic in the past. Here a malariometric survey was conducted using microscopy, RDT and real-time polymerase chain reaction (PCR) assays to screen malaria infections in asymptomatic carriers and to explore associated risk factors of residual malaria parasitaemia among local residents.

## Methods

### Study sites

**Fig. 1:**
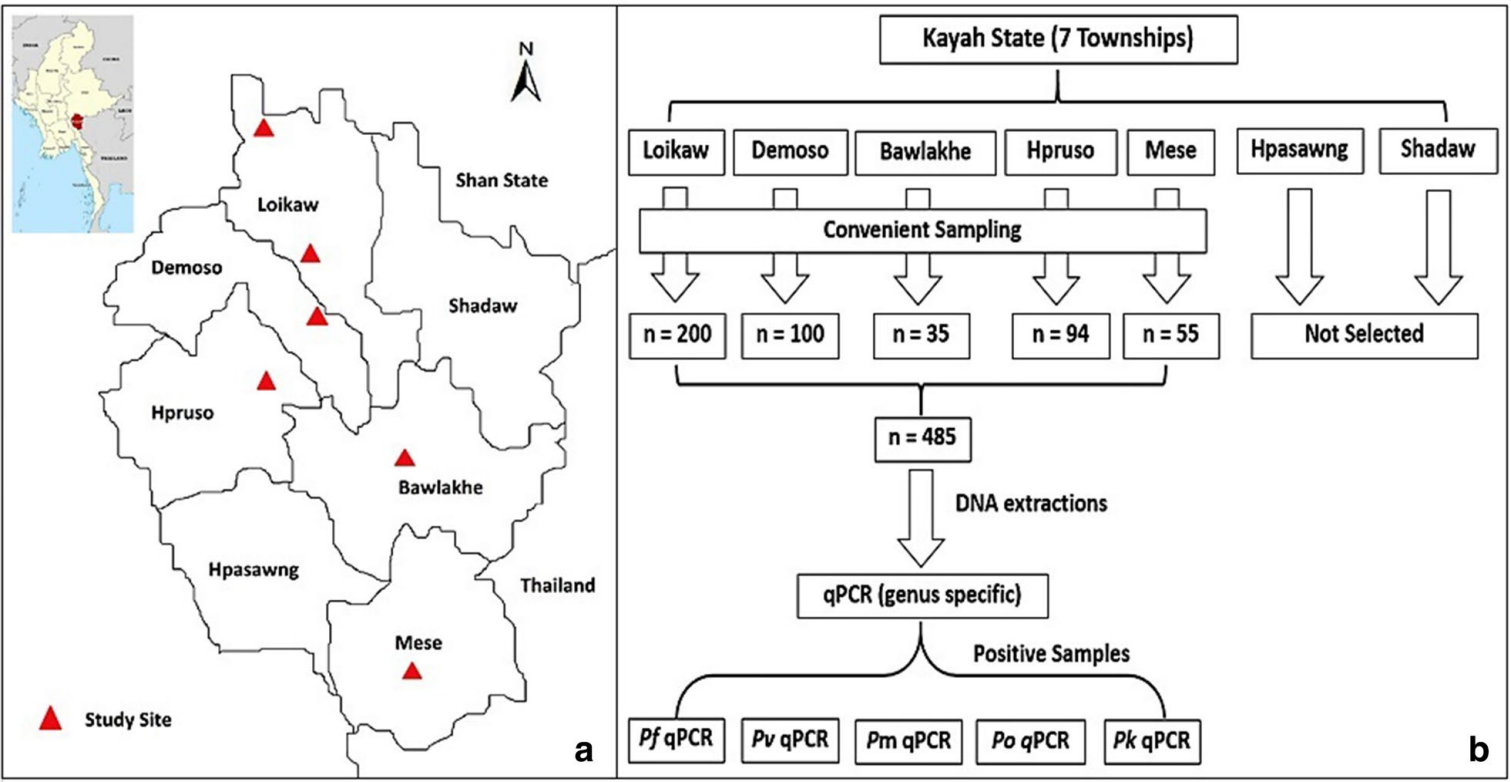
**a** Study sites in Kayah State, **b** steps of research procedure. n, number of samples; *Pf, Plasmodium falciparum*; *Pv, Plasmodium vivax*; *Pm, Plasmodium malariae*; *Po, Plasmodium ovale*; *Pk, Plasmodium knowlesi*

This study was conducted in Kayah State in eastern Myanmar, which borders Mae Hong Son Province in Thailand. Kayah has a tropical climate with average temperatures ranging from 16 °C in December to 30 °C in April. The rainy season is from May to late October with annual rainfall of approximately ~3000 mm. Malaria transmission is low, unstable and peaks from May to August, coincident with the rainfalls [19]. There are seven townships in Kayah State, five (Loikaw, Demoso, Hpruso, Mese, Bawlakhe) were chosen for the study (Fig. 1a). One village from each township (two villages in Loikaw Township) was selected to conduct a cross-sectional survey of malaria parasite prevalence.

### Data and sample collection

A community-based, cross-sectional study was conducted to determine the prevalence of asymptomatic *Plasmodium* spp. infections. The number of participants enrolled was based on the expected prevalence of malaria infection of 10% in Kayah State, according to the previous study in Myanmar [16]. Participants were recruited by convenience sampling (Fig. 1b). All individuals aged ≥6 months were invited to participate in the survey. Participants were interviewed using a pre-structured questionnaire, to collect general demographic and behavioural information including gender, age, occupation, education level, household size, usage and type of insecticide-treated bed nets, history of previous episodes of malaria, and treatment-seeking behaviour. For participants 5 years or younger, the interview was done with his/her parents instead. Symptom information was collected and the tympanic temperature measured for each participant. Finger-prick blood samples were used for RDT and microscopy. In addition, 200–300 μl of finger-prick blood from each available participant were collected into EDTA microtainers tubes and stored at −20 °C for subsequent molecular parasite detection by PCR.

### RDT

A CareStart™ Malaria HRP2/pLDH (Pf/PAN) Combo RDT was used to detect *Plasmodium* species in participant samples. This RDT uses histidine-rich protein 2 (HRP 2) to detect *P. falciparum* and lactose dehydrogenase (LDH) for *Plasmodium vivax, Plasmodium ovale* and *Plasmodium malariae.* This RDT has been shown to detect 99 and 94.3% of test samples with *P. falciparum* and *P. vivax*, respectively, at a density of 200 parasites/μl of blood and 100% of test samples with at a density of 2000 parasites/μl of blood for both species, with 1.5 and 0.7% false positive rates, respectively [20].

### Microscopy

Thick and thin blood smears were made on the same slide, air dried and transported to the laboratory of the 100-bed hospital in Loikaw Township. The slides were stained with 3% Giemsa for 10 min and screened for the presence of plasmodial infections. The slides were read by a local microscopist who was blinded to the individual RDT results. Parasitaemia and gametocytaemia were determined from thick blood films by counting the number of parasites per 200 white blood cells (WBCs). A slide was classified as negative if no *Plasmodium* asexual forms or gametocytes were found after counting 500 WBCs. Thin blood films were examined for *Plasmodium* species by counting the number of parasites per 5000 red blood cells (RBCs). For quality control purposes, a second experienced microscopist randomly selected 5% of the slides for re-examination.

### PCR

Whole blood samples, stored at −20 °C in microtainers were transferred on dry ice to the laboratory at the Faculty of Tropical Medicine, Mahidol University, Bangkok, Thailand. DNA extraction from 200 to 300 μl of each blood sample was carried out using the Favorgen 96-Well Genomic DNA Extraction Kit, following the manufacturer’s recommendations. Malaria parasite infection was first identified by the QMAL assay, a genus-specific probe-based quantitative PCR (qPCR) targeting a conserved region of the 18S rRNA genes of *Plasmodium* [21], by using 4 μl of purified DNA equivalent to 8 μl of whole blood. For positive samples by QMAL, 4 μl DNA was subjected to species-specific 18S qPCR assays to detect *P. vivax*, *P. ovale*, and *P. malariae* as previously described [22]. For *P. falciparum*, the *var*ATS qPCR assay was used [23]. *Plasmodium knowlesi* was detected with (forward primer: 5′-GTT AGC GAG AGC CAC AAA AAA GCG AAT-3′, reverse primer: 5′-ACT CAA AGT AAC AAA ATC TTC CAT A-3′, and hydrolysis probe: 5′-HEX-TGC TTT ATG TGC GCA TCC TCT ACC TA-BFQ-3′). All qPCR assays were performed with iTaq™ Universal Probe Supermix 2× on a CFX96 real-time PCR detection system.

### Statistical analysis

Data were checked for completeness and consistency, and entered into an SPSS 17.0 (SPSS Inc, Chicago, IL, USA) database. Descriptive statistics and differences in distributions were evaluated using the Chi square (χ^2^) test. An error probability (*P* value) of <0.05 was considered statistically significant.

### Ethical consideration

This study was approved by the Institutional Review Board of the Faculty of Tropical Medicine, Mahidol University (MUTM 2016-0140-01) and the Defense Services Medical Research Centre (DSMRC), Myanmar (IRB/2016/31). Written informed consent was obtained from all adults willing to participate in the study, and a parent provided consent for participants under 18 years of age. Malaria-positive participants were treated as per the national treatment guideline.

## Results

### Characteristics of the study population

A total of 485 samples were collected in this study, 54.4% (264) of which were from male participants. Adolescent and adult participants were predominant and only 8.6% (42/485) were children younger than 10 years old. The median age of participants was 32 years (6 months–65 years). More than 80% (420) of participants had a primary education or higher. The most frequent occupation among respondents (49.3%, 239) was forest-related work (farming, wood and bamboo cutting). More than 70% of respondents (377) had at least three family members in their household. Nearly all respondents reported using bed nets while sleeping at night and 18.1% (87) of bed nets used were insecticide treated. The remaining bed nets were ordinary bet nets (Table 1).

**Table 1:** Characteristics of participants

Characteristics	Number	Percent
Sex		
Male	264	54.4
Female	221	45.6
Age (years)		
<5	5	1
5–10	37	7.6
11–20	32	6.6
21–30	138	28.5
31–40	153	31.5
>40	120	24.7
Median (range)	32 years (6 months–65 years)	
Education		
No education	65	13.5
Primary to middle school	264	54.4
High school and above	156	32.1
Occupation		
Forest-related occupation	239	49.3
Students	54	11.1
Housemaker/dependent	192	39.6
Family size		
Less than 3 people	108	22.3
3–5 people	334	68.9
More than 5 people	43	8.9
Use of bed nets		
Yes	481	99.2
No	4	0.8
Type of bed nets (n = 481)		
Ordinary bed nets	394	81.9
Insecticide-treated bed nets	87	18.1

### Prevalence of malaria infection

The prevalence of malaria infection was detected by RDT, microscopy and qPCR. In this study, infections were considered asymptomatic when a participant’s body temperature was <37.5 °C at the time of blood collection and a participant reported no fever within the previous 2 days. The prevalence of *Plasmodium* infection was 1.03% (5/485; three by *P. falciparum*, one other species and one mixed) by RDT and 1.44% (7/485) by microscopy. Of the seven microscopy-positive samples, five were positive for *P. falciparum*, one for *P. vivax* and one for mixed (*P. falciparum and P. vivax*) infections.

Molecular screening by qPCR detected parasite DNA in 2.3% (11/485) of blood samples. *Plasmodium falciparum* accounted for 27.3% (3/11) and *P. vivax* for 72.7% (8/11). No mixed infection was detected by PCR. qPCR confirmed parasite DNA in 100% of RDT-positive samples and in 85.7% (6/7) of microscopy-positive samples. qPCR detected additional asymptomatic parasitaemia in six RDT-negative samples and five microscopy-negative samples. *Plasmodium ovale, P. malariae* and *P. knowlesi* were not found in this study.

Only one out of 11 participants with infections presented with fever >37.5 °C; this was confirmed as *P. falciparum* by PCR. The rest appeared to be healthy with no signs of malaria-like symptoms, including fever, chills and headache.

### Demographic characteristics associated with *Plasmodium* infections

A significantly higher prevalence of asymptomatic *Plasmodium* infections was found in males than females. The odds of being asymptomatic were about ten times higher among men than among women. Most qPCR-positive asymptomatic *Plasmodium* infections were found in adults. Among the positive samples, the youngest participant age was 17 years old. A higher prevalence of asymptomatic *Plasmodium* species infection was associated with forest-related occupations for malaria infection. Individuals who worked as farmers or wood and bamboo cutters had increased risk of having asymptomatic malaria infection, with an odds ratio approximately ten times greater than participants whose occupation were not forest-related. There were no statistically significant difference in infections by education, family size, or use and types of bed nets (Table 2).

**Table 2:** Association between characteristics and asymptomatic malaria infections

Characteristics	Total	PCR	Odds ratio (95% CI)	P value*
		Positive (%)		
Sex				
Male	264	10 (3.8)	8.67 (1.10–68.19)	0.014**
Female	221	1 (0.5)	1	
Age (years)				
>20	411	10 (2.4)	1.82 (0.23–14.44)	0.565
≤20	74	1 (1.4)	1	
Education				
Up to primary school	194	5 (2.6)	1.26 (0.38–4.18)	0.709
Middle school and above	291	6 (2.1)	1	
Occupation				
Forest-related	239	10 (4.2)	10.69 (1.36–84.24)	0.005**
Non-forest-related	246	1 (0.4)	1	
Family size				
Less than 3 people	108	3 (2.8)	1.32 (0.34–5.06)	0.687
3 people or more	377	8 (2.1)	1	
Use of bed net				
Yes	481	11 (2.3)	–	0.76
No	4	0 (0)		
Type of bed nets (n = 481)				
Ordinary bed nets	395	9 (2.3)	1.21 (0.26–5.53)	0.979
Insecticide-treated bed nets	86	2 (2.3)	1	

* P value by Chi square test

** Significant at *P* < 0.05

## Discussion

Most countries in the GMS, including Myanmar, are currently at the pre-elimination phase of malaria. Timely identification and prompt treatment of symptomatic malaria infections, as well as detection of asymptomatic individuals and treatment of malaria reservoirs, are deemed essential for eliminating the source of disease transmission. Information about the epidemiology of asymptomatic malaria infection in Myanmar is sparse. This study provided additional data on the prevalence of asymptomatic malaria infections in Kayah State, eastern Myanmar.

Microscopy is currently the gold standard for malaria diagnosis among symptomatic patients. However, low-density infections especially among asymptomatic parasite carriers, are easily missed by microscopy diagnosis because parasite densities are often below the detection limit of microscopes (normally above 50 parasites/μl of blood) [24–27]. In this study, individuals with low parasitaemia were more likely to be missed by the local microscopist. Several studies have indicated that low-density infections are more easily missed by light microscopy [12, 16, 17]. Similar discrepancies in malaria diagnosis have been reported for recent studies conducted in the same region as in this study [11, 17].

Because of generally lower parasitaemia in asymptomatic infections, a more sensitive test is critical for identifying asymptomatic carriers. Using PCR, asymptomatic *P. falciparum* and *P. vivax* infections were identified. The prevalence was similar to the values (1.9%) found in a previous study conducted in Mae Hong Son, Thailand, which was also determined by PCR [13]. The result of the current study was also within the range of asymptomatic malaria infection in Myanmar, which has been reported to be 1.0–9.4% in southern areas [16]. *Plasmodium vivax* was the most prevalent species among asymptomatic cases in this study, similar to the distribution of malaria species reported in other works [11, 12]. However, a recent study in central Myanmar reported the low prevalence of asymptomatic malaria infection, with a predominance of *P. falciparum* [28]. The difference likely reflects the heterogeneity of malaria species distribution in central Myanmar.

Age is considered one of the most important factors that correlate with protective immunity in malaria-endemic areas [9]. Young children are generally the most vulnerable to developing the disease. However, adults and older children who might have had several episodes of malaria and have acquired immunity are more likely to harbor asymptomatic infection [29, 30]. This study found that all asymptomatic infections were restricted to participants aged >17 years. Probably due to the low overall prevalence (2.3%) and the small number of children enrolled in this study, the difference in infection rates between adults and children did not reach statistical significance [7]. That only adults were infected suggests that these infections were more likely transmitted outside of residential areas, consistent with forest-related activities being a strong risk factor. Entomological data for local vectors would be helpful in establishing the most likely areas of active transmission. This finding is also consistent with the lack of association of malaria infections with the use of bed nets.

Many previous studies have found that males are more likely to have *Plasmodium* infections than females [14, 31–33]. In this study, men working in the forests had much higher infection rates. Because men tend to engage in more agricultural and forest-related activities, such as wood and bamboo cutting as similarly reported in another study [34], the risk was most likely occupational. When occupational risk factors had been taking into account, the difference in infection rates between male and female was no longer statistically significant.

This work is among a very few studies [4, 16] reporting the current situation of malaria in Myanmar, whose mountainous terrain limits access to local communities in many areas. This cross-sectional study was conducted in June, and thus the seasonal pattern of asymptomatic malaria infection cannot be assessed. However, the available data clearly indicate that the study areas are currently at the low end of endemicity. These data provide justification for investing resources to target male forest workers, so as to interrupt malaria transmission.

## Conclusion

A combination of RDT, light microscopy and PCR diagnostics was used to identify asymptomatic malaria infections. Risk factors were identified to improve malaria risk stratification and to guide decision-makers towards better design and delivery of targeted interventions in small villages, representative of Kayah State. In addition to the routine statistics on symptomatic cases, information on asymptomatic cases is useful for determining the true burden of disease in the area. Further studies, including entomological aspects, genotyping of asexual and sexual parasites, and immune system evaluation, are needed to better understand the epidemiology of asymptomatic *Plasmodium* spp. infections and their contribution to the dynamics of malaria transmission and to the incidence of symptomatic infections.
